# A General Effort

**Published:** 1921-04-16

**Authors:** 


### A GENERAL EFFORT.

The idea of a joint effort on the part of the voluntary hospitals to secure the notice of the public to their aims and needs generally is not a new one. The Red Cross Society has thought of it, tried it, and not yet succeeded. The Committee of Management of the Great Northern Central Hospital, the publicity and appeal methods of which are far from sluggish, consider that, "in view of the general public's limited opportunities of gaining knowledge of voluntary hospital questions, an effort should be made to form a Voluntary Hospitals' Publicity and Propaganda Committee, towards the funds of which all hospitals should be invited to subscribe according to their number of beds and out-patients. Such a Committee should consist of hospital representatives, with some of the best publicity experts."

The suggestion is that this Committee would endeavour to promote the interests of voluntary hospitals generally, and leave it to individual institutions to make known their particular needs. That is to say, that the ground-bait would be laid down by the Committee and the individual hospital would do the angling. Most of us are familiar with the advertising methods of a central gas association, for instance, which by its cleverly worded and illustrated notices inculcates a general belief that a gas-fire can be so excellent that nobody, however perfect are his eyes, ears, and nose, will detect that it is a gas-fire. Then we have a group of health resorts advertising themselves collectively as a Riviera and organising a public opinion that the weather is always perfect in their part of our island. This form of advertising would not be continued during many years if it did not bring about results.

Concerted work in appeal and propaganda was also strongly urged at the Annual Meeting of the Manchester Children's Hospital by Mr. Frank H. Roby, an account of which will be found on another page.

The Hospital likes the scheme proposed by the Great Northern Hospital, because in itself it has been such a scheme for a great many years and is, as always, very much at the command of the voluntary hospitals to make known to the general public the work and needs of these institutions.

